# Deciphering immune microenvironment and cell evasion mechanisms in human gliomas

**DOI:** 10.3389/fonc.2023.1135430

**Published:** 2023-05-19

**Authors:** Soumaya Rafii, Sarah Kandoussi, Amina Ghouzlani, Oumayma Naji, Konala Priyanka Reddy, Rizwan Ullah Sadiqi, Abdallah Badou

**Affiliations:** ^1^ Immuno-Genetics and Human Pathologies Laboratory, Faculty of Medicine and Pharmacy, Hassan II University, Casablanca, Morocco; ^2^ Faculty of Medicine, Medical University of Pleven, Pleven, Bulgaria; ^3^ Faculty of Science and Technology, Middlesex University, London, United Kingdom; ^4^ Mohammed VI Center for Research and Innovation, Rabat, Morocco and Mohammed VI University of Sciences and Health, Casablanca, Morocco

**Keywords:** glioma, immune microenvironment, immune checkpoints, glioma cell evasion, therapy resistance, immunotherapy

## Abstract

Gliomas are considered one of the most malignant cancers in the body. Despite current therapies, including surgery, chemotherapy, and radiotherapy, these tumors usually recur with more aggressive and resistant phenotypes. Indeed, the survival following these conventional therapies is very poor, which makes immunotherapy the subject of active research at present. The anti-tumor immune response could also be considered a prognostic factor since each stage of cancer development is regulated by immune cells. However, glioma microenvironment contains malignant cells that secrete numerous chemokines, cytokines and growth factors, promoting the infiltration of immunosuppressive cells into the tumor, which limit the functioning of the immune system against glioma cells. Recently, researchers have been able to reverse the immune resistance of cancer cells and thus activate the anti-tumor immune response through different immunotherapy strategies. Here, we review the general concept of glioma’s immune microenvironment and report the impact of its distinct components on the anti-tumor immune response. We also discuss the mechanisms of glioma cell evasion from the immune response and pinpoint some potential therapeutic pathways, which could alleviate such resistance.

## Introduction

Gliomas are considered the most common malignant brain tumors, representing 75% of malignant central nervous system tumors, with a worldwide incidence of 6 per 100.000 person-years (1). These constitute a profound and unsolved clinical problem. Even though considerable progress has been made in treating other types of cancers, many questions remain unanswered in the case of gliomas (2). We know very little about the factors related to their appearance and their dynamic of evolution (3). Despite current therapies, including surgery, chemotherapy, and radiotherapy, these tumors usually recur with more aggressive and resistant phenotypes (4). Patients live an average of 15 months, and less than 5% of them are alive at five years. The most common glioma in adults is astrocytoma that includes glioblastoma (GBM), which appears to be the most aggressive brain tumor diagnosed in adults, showing abysmal prognosis (5).

Clinical signs of gliomas are very variable. These can range from headaches, ocular changes, or gastrointestinal manifestations such as loss of appetite, nausea, and vomiting. Changes in personality, mood, mental capacity and concentration might be observed as well (6). Furthermore, the prognosis of patients with gliomas is directly linked to the tumor’s degree of differentiation and malignancy and, thus to the grade (7). Since each stage of cancer is regulated by immune cells, the anti-tumor immune response could be used as a prognostic factor (8).

The present review aims to describe the immune microenvironment within glioma. We will discuss the involvement of the immune system in the regulation of glioma growth and pinpoint some potential therapeutic pathways, which could enhance the immune system to fight against glioma.

## General concept of immune microenvironment in glioma

Initially, the brain was considered to have no immunosurveillance activity and was, therefore, considered an “immunologically privileged” site. This view was largely accepted because of the presence of the blood-brain barrier (BBB) and the anatomical segregation of the brain from general circulation. It was thought that the brain did not have draining lymphatics, and microglia were considered the main antigen presenting cells in the brain tumor microenvironment (9). However, in 2015, Louveau et al. discovered functional lymphatic vessels lining the dural sinuses while trying to find out about T-cell gateways into and out of the meninges. These structures express all of the molecular hallmarks of lymphatic endothelial cells. They can carry both fluid and immune cells from the cerebrospinal fluid and are connected to the deep cervical lymph nodes (10). Furthermore, these authors showed, using immunohistochemical analysis, that the meningeal lymphatic vessels could carry leukocytes (10).

In the brain, the innate immune system is the first line of defense against pathogens and includes phagocytes, such as microglia and granulocytes, like neutrophils. The adaptive immunity includes T and B lymphocytes, which will induce immune memory for later dangers (11). Innate and adaptive immunity interact closely to maintain a good immune balance within the brain (9).

The glioma microenvironment contains malignant cells that secrete numerous chemokines, cytokines and growth factors that promote infiltration of various cells into the tumor, including astrocytes, pericytes, endothelial cells, circulating progenitor cells and Treg cells (12). There are also many immune cells, such as microglia, peripheral macrophages, myeloid-derived suppressor cells (MDSC), leukocytes and CD4+ T cells (12).

## Innate immune response in glioma

**Figure 1:**
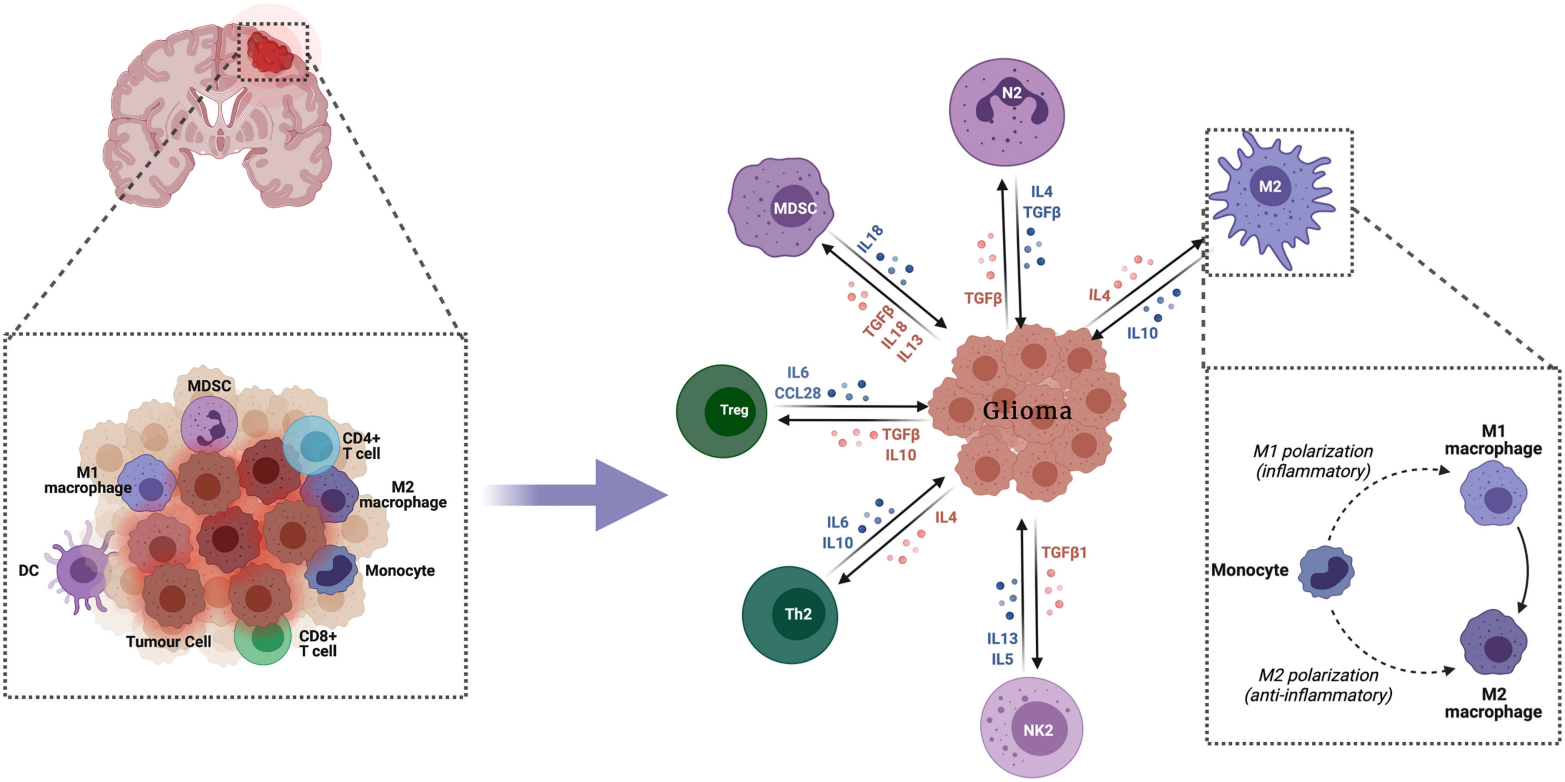
Innate immune response in glioma. The innate immune system comprises numerous hematopoietic cells, which collaborate with each other and with the adaptive immune system to enhance immunity, inflammation and tissue repair.

The innate immune system comprises many hematopoietic cells cooperating and with the adaptive immune system to promote immunity, inflammation and tissue repair (13) (**Figure 1**).

### Microglia or glioma-associated macrophages

Microglias are myeloid cells which reside in the CNS. These constitute the most abundant immune cells in brain tumors often representing up to 30% of the tumor mass (14). In gliomas, microglia are mainly polarized towards the M2 phenotype and do not express co-stimulation molecules that are essential for the full activation of T lymphocytes (15). Their capacity to regulate the expression of MHC class II molecules positively is altered and hence express immunosuppressive ligands such as PD-L1 on their surface. The secretion of pro-inflammatory cytokines such as IL1β, IL-6, and TNFα, which are essential for developing an efficient innate immune response, is also decreased (16). Also, the reduction of microglia polarization by P4H (a necessary enzyme for procollagen hydroxylation and collagen synthesis and secretion) induces inhibition of proliferation and invasion of GBM cells (17). Clinical studies have shown significant infiltration of gliomas associated macrophages (GAM) in tumors. In fact, their numbers increase with the degree of malignancy (12). Monocytes recruited to the tumor site have a high expression of CCR2, which decreases as they differentiate into macrophages (18). Chen et al. observed a broad range of CCR2 expression in the GAM, suggesting an active transformation from infiltrating monocytes to mature macrophages. These are mainly found around blood vessels and then spread in the tumor. It may imply that these GAMs interact with neoplastic cells in the perivascular area to promote tumor development. The authors also found that GBM patients who had a low expression of CCL2 (monocyte chemoattractant protein) had a significant prolonged survival, inhibiting the CCL2-CCR2 axis in glioblastoma-bearing mice significantly increased their survival (19).

Periostin (POSTN) is a tumor-associated macrophages (TAM) attractant that is preferentially secreted by glioma stem cells (GSCs). In this study, Zhou et al. found that its protein level positively correlates with TAM density in primary GBM. The authors used two specific shRNA to silence POSTN in GSCs and significantly reduced its chemoattractant effect. Furthermore, when disrupting POSTN in the *in vivo* model, it significantly reduced the recruitment of the TAMs, especially those of the M2 phenotype and inhibited GBM tumor growth (20). Another study demonstrated that glioma cells with heterozygous IDH1 R132H mutation change TAMs towards a phagocytic anti-tumor phenotype (21).

### Natural killer cells

Natural killer (NK) cells are effector lymphocytes that recognize and lyse target cells that display the MHC I or MHC I-like proteins. The NK group 2D (NKG2D) receptor is an activating NK and CD8 T-cell receptor that mediates cytotoxicity by ligating stress-inducible ligands on target cells (22). Some studies reported that the tumor-infiltrating NK cells were non-functional, likely due to contact with immunosuppressive cells such as GAM and regulatory T cells, which might inhibit the secretion of IFN-γ by NK cells by producing TGFβ (23). Tumor cells may inhibit NK cell function by releasing TGFβ and IL10, although in some cases, IL-10 has also been shown to activate NK cells (24). On the other hand, Zhang et al. have reported that the IDH mutant glioma stem-like cell lines have significantly lower expression of NKG2D ligands compared with IDH wild-type cells (25). It suggests that the IDH mutant glioma cells acquire resistance to NK cells through epigenetic silencing of NKG2D ligands ULBP1 and ULBP3 (25). As a therapeutic strategy, the authors used the hypomethylating agent 5-aza-2’deoxycytodine (decitabine). It was found to restore ULBP1 and ULBP3 expression in IDH mutant glioma cells (25). Another study generated a CAR NK cell line based on the human NK cell line KHYG-1. The newly generated EvCAR-KHYG-1 cell line inhibited GBM cell growth via apoptosis in EGFRvIII expressing cells, in a specific manner. Thus, EvCAR-KHYG-1 may become an effective treatment option for patients with GBM (26).

### Dendritic cells

This heterogeneous population of professional antigen-presenting cells (APC) is an essential link between innate and adaptive immunity. These are specialized in presenting antigens to T and B cells to initiate adaptive immunity or immunological tolerance. In the case of gliomas, there is a complex interaction between DCs, microglia, T lymphocytes, and tumor cells in the tumor microenvironment (TME) (11). Their role is the presentation of tumor antigens in the brain or cervical lymph nodes to initiate responses by effector T lymphocytes associated with immune defense, such as cytotoxic T lymphocytes (CTL) and CD4+ T lymphocytes (27). Garzon-Muvdi et al. reported that treatment with a TLR3 agonist leads to DC activation and increased infiltration of T effector cells in the tumor, in addition to a decrease in tumor-infiltrating Tregs. The authors further showed that treatment with TLR3 agonist in addition to anti-PD-1 blockade improved survival in the preclinical orthotopic GBM mouse model (28). These observations indicate that the activation of antigen presentation provides an effective way to boost the antitumor immune response in glioma patients.

### Myeloid-derived suppressor cells

These are immature myeloid cells that promote the vascular supply of the tumor and disrupt main immunosurveillance mechanisms. Studies have shown that their level increases in the blood of patients with glioblastoma (29). Their origin is unknown. However, normal monocytes cultured with glioma cells acquire MDSC-like properties such as increased production of immunosuppressive factors, suggesting that MDSC could originate from glioma infiltrating-monocytes (30). A study revealed that the Polymorphonuclear (PMN) MDSC that expressed lectin-type oxidized LDL receptor 1 (LOX-1) inhibited T cell proliferation and enhanced immune suppression, which may play a key role in driving GBM progression (31). In addition, Guo et al. reported that gliomas-derived exosomes (GDEs) play an important role in MDSC differentiation and facilitate their expansion and function in hypoxic conditions. It promotes the upregulation of miR-21 and miR-10a expression in GDEs, leading to MDSC activation via Rora/IκBα/NF-κB and Pten/PI3K/AKT pathways that are involved in glioma progression (32) One study showed that PI3K inhibition had the most substantial effects on global signaling pathways implicated in glioma expansion, reducing tumor cell proliferation (33). The GBM microenvironment contains an accumulation of the monocytic subset of MDSCs (M-MDSCs) which expressed high levels of the MIF cognate receptor CD74. However, blocking M-MDSCs with Ibudilast, an inhibitor of MIF-CD74 interaction, reduced MDSC function and enhanced CD8 T cell activity in the tumor microenvironment (34).

## Adaptive immune response in glioma

The primary response is generated by circulating B and T lymphocytes, which have established specificity for a given antigen. When they bind to an antigen recognized by the B cell receptor, B lymphocytes proliferate and secrete antibodies specific to this pathogen. T cells, on the other hand, need Interaction of the TCR with peptide-MHC. T cells are subdivided into two main groups (12, 35):

### B cells

B cells contribute to immunity by producing antibodies and presenting antigens (36). Another important strategy B cells use to influence immunological responses in the central nervous system is the release of pro-inflammatory and anti-inflammatory cytokines. Anti-inflammatory cytokines are secreted by regulatory B (Breg) cells, whereas pro-inflammatory cytokines are secreted by antigen-experienced B cells (36, 37). Glioblastoma promotes the transformation of B cells into Breg cells, which maintains tumorigenicity (38). According to one study, Bregs accounted for 10% of bone marrow-derived infiltrating immune cells in two orthotopic brain tumor models, and 40% of GBM patients who were examined tested positive for B-cell tumor infiltration. GBM-associated B cells suppressed activated CD8+ T cells, as evidenced by the expression of inhibitory molecules PD-L1 and CD155 and the production of immunoregulatory cytokines TGF and IL10 (39). Extensive animal survival after local administration of B cell-depleting immunotherapy emphasized the pathophysiologic significance of B cells (39). Based to studies, B cells expressing the costimulatory marker 4-1BBL (or CD137L) can boost CD8+ T cell antitumor cytotoxicity (40). To sustain the Ag presentation function of B cells (41), 4-1BBL+ B cells were activated utilizing CD40 and IFN receptor (IFNR) ligation (designated as BVax). BVax migrates to critical secondary lymphoid organs and is adept at antigen cross-presentation, which increases CD8+ T cell survival and functioning (41). A combination of radiation, BVax, and PD-L1 inhibition resulted in tumor elimination in 80% of tumor-bearing rats treated. This treatment induced immunological memory, which prevented the formation of new tumors in cured animals upon reinjection (41).

### CD8+ T and CD4+ T cells

CD8+ T cells are activated upon antigen presentation through MHC class I molecules. This T cell subtype constitutes a crucial component of the tumor-specific adaptive immunity, which differentiates into cytotoxic T lymphocytes, directly lysing target cells (42). Han et al. showed that the infiltration of CD8+ T cells in tumors inversely correlated with glioma grades and could be a predictor of clinical outcome (43). Besides, Yang et al. have found that miR-15a/16 defficiency increased tumor-infiltrating CD8+T cell number and enhanced CD8+T cell-mediated immune response via targeting mTOR. The authors identified the target gene (mTOR) by a computer-assisted analysis and reported a direct interaction between miR-15a/16 and mTOR but not with other genes. This was also closely associated to T cell function (44). The authors also showed that GL261-bearing mice deficient in miR-15a/16 had lower expression of PD1, TIM3 and LAG3 and displayed higher production of IFNγ, IL2 and TNFα, which relieved the immunosuppressive state of CD8^+^ T cells, reduced the tumor volume and prolonged mice survival (44).

CD4+ T cells detect mainly extracellular proteins recognized through MHC class II receptors on APCs (45). This T cell subset mediates systemic immunity, compatible with long-term tumor eradication (46). CD4+ T cells are central in initiating and maintaining anti-cancer immune responses. Nonetheless, CD4+ regulatory T cells (Tregs) suppress anti-tumor immunity and promote tumor progression (43). It was demonstrated that when CD4+ T cells with tumor-specific TCRs were administered to tumor-bearing mice, it mediated direct cytotoxicity against tumor cells (47). Wang et al. demonstrated that CD4+ CAR T cells mediated CD8-independent GBM eradication with long-term efficacy both *in vitro* and *in vivo*. This finding pinpoints the importance of the CD4+ cell subset for effective CAR therapy (48).

In gliomas, the infiltrating CD4+ and CD8+ cells represent a low percentage compared to GAM (49). T lymphocyte cell numbers decrease in low-grade glioma compared to high-grade (50). The lack of adequate activation of T lymphocytes in the tumor microenvironment may be because anti-tumor responses of T lymphocytes are inhibited by soluble factors such as TGF-β and IL-10 that are secreted by glioma cells (12). Besides, glioma cells lack the co-stimulation molecules B7.1/2, and overexpress the PD-L1 mRNA and protein, a potent inhibitor of CD4+ T cell functions (51). Previous studies showed that high-grade gliomas exhibit high levels of CD4+ but low levels of CD8+ TILs. This might be another reason behind the compromised immune function in the high-grade glioma tissues (43).

Regulatory T cells are considered to be CD4+ CD25+ FoxP3+ T cells. Their natural role is to prevent the immune response from causing significant damage to internal tissues (52). In glioma, it is now well established that tumors infiltrating Treg cells are associated with a poor clinical prognosis (53). The tumor makes use of the immunosuppressive functions of Treg cells to escape immune responses. It would be interesting to attenuate local and specific Treg cells for glioma treatment to improve anti-tumor immune responses (54). Zhang et al. showed that down-regulation of FoxP3 promotes glioma cell growth and suppresses its apoptosis, while its up-regulation inhibits the invasion ability of glioma cells and suppresses migration (55). It was also reported that FoxP3 overexpression favors apoptosis of glioma cells; it also considerably enhances the induction of apoptosis by TNF-a or chemotherapeutics (56). A transcriptomic study showed that Foxp3 expression increases with the aggressiveness of gliomas and is associated with poor survival in TCGA and Moroccan patients (57). **Table 1** summarizes some of the immunological differences between low-grade and high-grade gliomas.

**Table 1:** Comparison of immune cells between GBM and LGG.

	Comparison of immune cells between GBM and LGG
**Myeloid cells**	• GBM favors MDSCs remaining as MDSCs (58). • LGG promotes MDSCs maturing to DCs (58). • GBM have a significant infiltration of glioma-associated macrophages (12). • The level of M0 gradually increases from LGG to GBM (59).
**APC and NK**	• Reduced antigen-presenting cells and NK cells are found in GBM patients, which are an indication of a diminished anti-tumor response (58).
**T lymphocytes**	• T lymphocyte cell numbers decrease in low-grade glioma compared to high-grade (50). • Treg infiltration was found to be positively associated with the grade of the glioma tumor (57). • Infiltration of CD8+ T cells in tumors inversely correlated with glioma grades (43).

## Mechanisms of glioma cell evasion from the immune response

### Immune checkpoints

Immune checkpoints are a group of co-stimulation and co-inhibition pathways that limit the functioning of the immune system. Recently these have been the subject of extensive research. Through the blockade of inhibitory immune checkpoints, researchers could reverse immune resistance of cancer cells and thus activate the anti-tumor immune response (60, 61).

### Programmed death-ligand 1

PD-L1 has significant involvement in immune evasion in GBM. It inhibits the proliferation and function of cytotoxic T lymphocytes and promotes the activity of Treg cells by binding to Programmed cell death 1 (PD-1). The expression of PD-L1 on tumor cells and T lymphocytes is correlated with tumor grades and poor overall survival of glioblastoma patients (62). Microglia and TAM are also known to express PD-L1 on their surface and simultaneously promote its expression on GBM cells, which make this immune checkpoint a prime target for immunotherapy for these patients (63).Blochet al. showed that the expression of PD-L1 on glioma-infiltrating macrophages is upregulated by glioma tumor cells, which made these macrophages capable of suppressing T cell activity through IL-10 signaling. Inhibition of IL-10 and IL-10 receptor reduced the expression of PD-L1 on monocyte by more than 50% (51). In hypoxia, there is an increased expression of PD-L1 in glioma cell lines. In addition, PD-L1 and HIF-1α inhibitor therapy reduced tumor mass and improved dendritic cell (DC) and CD8+ T cell activation in a mouse model of glioma (64). PD-L1 is also correlated to IgSF11, VISTA and CTLA-4 in high-grade glioma (65).

### The programmed cell death protein 1 receptor

PD-1 is expressed on activated CD8^+^ T cells, as well as B cells and natural killer cells, in the event of chronic antigen exposure. By binding to its ligand, it leads to inhibition (66). It is also expressed on suppressive myeloid cells in human GBM. However, combined blockade of PD1 and TIGIT restored antigen specific T cell proliferation that is inhibited by MDSC (67).

Zhao et al. confirmed that GBM patients responsive to anti-PD1 immunotherapy had significantly better overall survival after treatment (68). However, tumors from non-responders had significantly more PTEN mutation than the responsive ones and were associated to immunosuppression signatures such as Treg cells (68). In another study, it was shown that combining Anti-CXCR4 and anti-PD-1 on mice that were implanted with glioma cells, presented immune memory and decreased populations of immunosuppressive tumor-infiltrating leukocytes within the brain (69). This treatment also improved CD4+/CD8+ ratios in the brain and contributed to increased levels of pro-inflammatory cytokines (69).

### Cytotoxic T-lymphocyte antigen

CTLA-4 is expressed on activated CD4+ and CD8+ T lymphocytes and Treg cells. It plays a major role in the escape of gliomas from the immune system (70). Several studies demonstrated that the expression of CTLA-4 is positively correlated with disease progression in patients with glioma. Meanwhile, blocking it, increased the proliferation capacity of CD4+ T cells (71). This suggests that targeting it could perhaps increase the anti-tumor activity of T cells by the up-regulation of granzyme B (71). Selby et al. found that anti-CTLA-4 induced the loss of intratumoral Tregs along with the expansion of CD8 T effector in mice (72).

### T cell immunoglobulin and mucin domain-containing protein 3

TIM-3 is expressed on activated T cells, NK cells, and monocytes. It binds to its ligand Gal-9, which plays an essential role in tumor survival, and even the progression of various malignant tumors; however its role in tumorigenesis is still unknown (73). In patients with glioma, the expression of TIM-3 on CD4+ and CD8+ T cells is significantly higher than in healthy controls, and its expression level correlates with the grade. Similarly, the use of monoclonal antibodies to block TIM-3 increases the proliferation of T cells (74). Kim et al. investigated TIM-3 expression in a glioma model and the antitumor efficacy of TIM-3 blockade. These authors demonstrated that anti-TIM-3 mAb alone had no significant improvement in a mouse model *in vivo*. However, triple therapy with anti-TIM-3, anti-PD-1, and stereotactic radiosurgery resulted in overall survival of 100% (75).

## Immunotherapy

For gliomas, treatment often involves surgery, radiation therapy, and chemotherapy. Surgical removal generally improves neurological functions and reduces dependence on corticosteroids (76). Patients are then treated by external radiotherapy or chemotherapy. However, survival following conventional therapies is very poor (77), which makes immunotherapy the subject of active research at present.

### Vaccines

#### Dendritic cell vaccines

**Figure 2:**
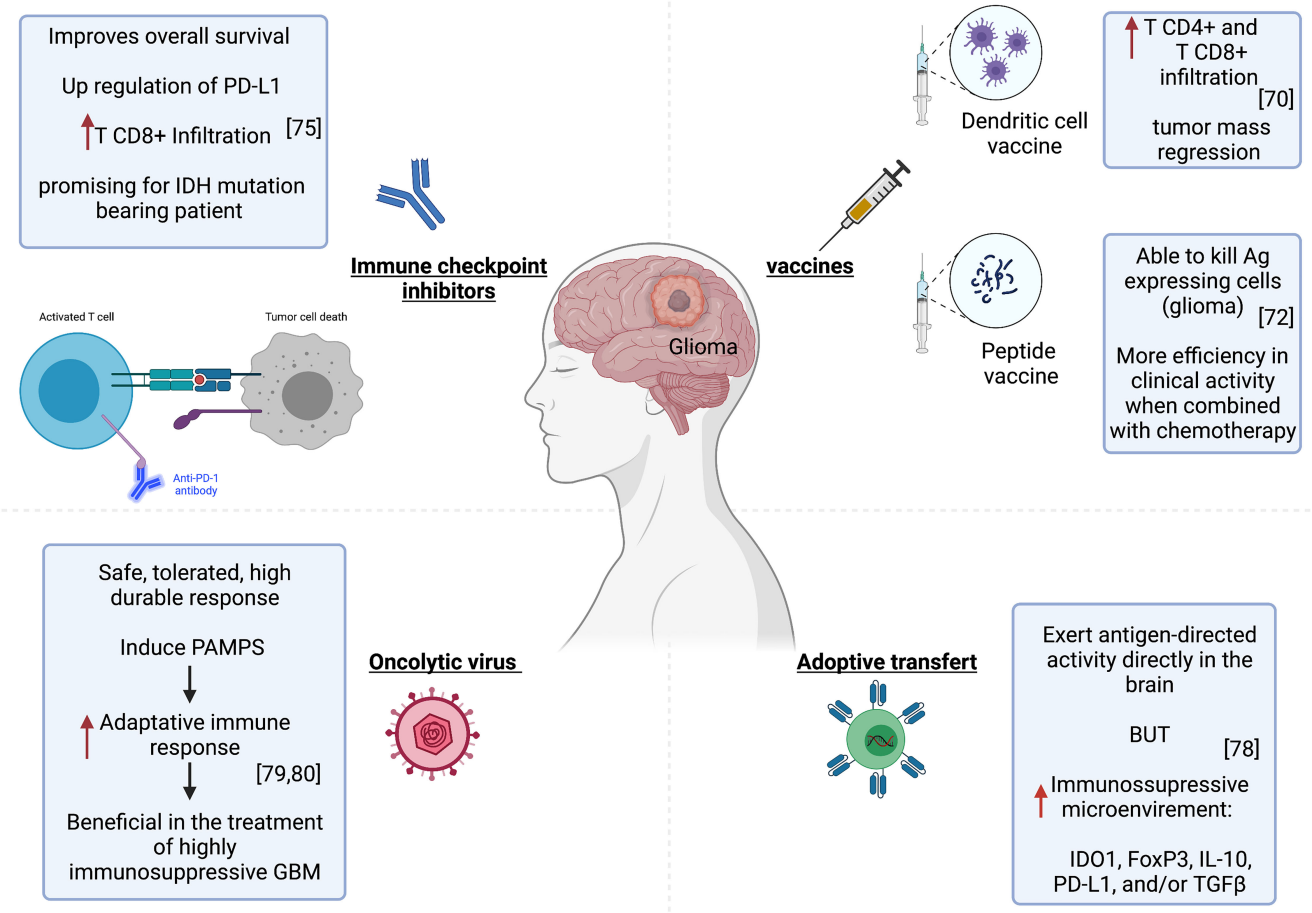
Immunotherapy strategies in glioma. Left to right, improved patient survival and tumor regression associated with successful CD4+ and CD8+ T infiltration after treatment with Pembrolizumab or DC-based immunotherapy. The tumor microenvironment became immunosuppressive upon CART cell injection. Oncolytic virotherapy can provide a potent stimulus to the immune system and activate specific anti-tumor immunity.

Dendritic cell vaccines constitute one of the most explored immunotherapies in glioblastomas. These are made by exposing DCs, which are taken from patients to tumor peptides or tumor mRNA. These cells are then transfused to the patient to activate the immune response (78). In a preclinical study, Garg et al. used a single-agent immunogenic cell death (ICD) inducer–based DC vaccine in an orthotopic High Grade Glioma (HGG) mouse model and tested its efficacy as a next-generation anti-HGG immunotherapy. In prophylactic setups, the ICD-based DC vaccines showed considerable survival benefit against HGG, especially in combination with standard chemotherapy Temozolomide (TMZ). It increased median survival by more than 30%. However, the efficacy of this vaccine was highly dependent on an intact adaptive immune system and a high number of CD8+ T cells. It also induced a high infiltration of TH1/CTL/TH17 cells and reduced Treg (79). In human subjects, the DC-based immunotherapy strategy appears promising for inducing anti-tumor immune responses. Treated patients had developed significantly more CD4+ and CD8+ T cell infiltrations in the tumor compared to the pre-vaccination state. Furthermore, the magnetic resonance imaging showed a regression of the tumor mass after vaccination (80) (**Figure 2**).

#### Peptide vaccines

These are based on the induction of an immune response by injecting tumor-specific antigens, which are absent from normal tissue. These antigens are often proteins encoded by mutant genes in the tumor. These are relatively conserved among different types of cancers and patients (81). Shibao et al. evaluated VEGFR1 and VEGFR2-derived peptides in vaccines targeting tumor vasculatures in high grade gliomas. VEGFR-specific CD8+ T cells induced by the vaccine were able to kill not only VEGFR-expressing endothelial cells but also glioma (82) (**Figure 2**). Furthermore, the combined application of this peptide vaccine and bevacizumab (anti-VEGF) exhibited more efficacy in clinical activity for high grade glioma patients (82). A clinical trial has shown that a vaccine targeting the IDH1 mutant in patients with grade III or grade IV glioma elicited immune responses in both T and B cells (83).

### Immune checkpoint inhibitors

An increasing number of clinical trials have been underway since 2011 to assess the potential therapeutic efficacy of PD-1/PD-L1 and CTLA-4 inhibitors as monotherapies and combination therapies for GBM (71). In one study, the immune analysis indicated that pembrolizumab anti-PD-1 monotherapy couldn’t induce an effector immune response in most GBM patients, likely because of immune-suppressive CD68+ macrophages preponderance in the tumor microenvironment and also insufficient T-cell frequency within the tumor microenvironment to eliminate the tumor (84). However, Cloughesy et al. have found that pembrolizumab confers significant improvement in the overall survival of patients with recurrent glioblastoma. The treatment is associated with an upregulation of PD-L1 and CD8+ T cell infiltration in tumors (85) (**Figure 2**). Another ongoing clinical trial is being conducted on IDH1 or IDH2 mutation-bearing adult glioma patients to test if nivolumab (anti- PD-1 antibody) stops tumor growth and provides long-lasting control of the tumor. This nivolumab-based treatment proved indeed promising for IDH-mutation-bearing glioma patients (NCT03718767) https://clinicaltrials.gov/ct2/show/NCT03718767.

### Adoptive transfer

T lymphocytes are carefully designed to express chimeric antigen receptors (CARs specific to the tumor. The interest of this approach lies in the capacity of CAR T cells to have MHC-unrestricted recognition of target cells by using antibody (ab) binding regions that allow T cells to react to epitopes formed by proteins, carbohydrates and also lipids. This would overcome many mechanisms by which tumors avoid immunorecognition, such as MHC down-regulation (86). This approach has shown promise for the treatment of glioblastomas (87). O’Rourke et al. conducted the first-in-human clinical trial of CAR-modified T cell (CART)–EGFRvIII in patients with recurrent GBM expressing EGFRvIII. The CART cells infiltrated the brain tumor and exerted antigen-dependent activity. However, the tumor microenvironment became immunosuppressive upon CART cell injection, with the expression of many immunosuppressive molecules, particularly IDO1, FoxP3, IL-10, PD-L1, and/or TGFβ (88) (**Figure 2**).

### Oncolytic viruses

Oncolytic virotherapy relies on replicating viruses that can selectively kill infected cancer cells (89). It can be a powerful stimulus for the immune system and lead to activating specific anti-tumor immunity (89). Besides, the innate immunity induced by the pathogen associated molecular patterns (PAMPs) can act as a powerful adjuvant to enhance the adaptive immune response. The use of this immunotherapy would be particularly beneficial in the treatment of highly immunosuppressive tumors such as GBM (89, 90)(**Figure 2**). Virotherapy for malignant gliomas has proven safe. Furthermore, Andtbacka et al. successfully used the talimogene laherparepvec, an oncolytic herpes simplex virus armed with granulocyte-macrophage-colony-stimulating factor (GM-CSF). It is the first oncolytic immunotherapy to demonstrate therapeutic benefit against melanoma in phase III clinical trial. It was well tolerated and resulted in a higher durable response rate (DRR). This gives hope for virotherapy success in the treatment of neoplastic diseases (91).

## Mechanisms of resistance to glioma treatments

Numerous metabolic stresses, such as hypoxia, acidosis, stem cells and blood-brain barrier, are present in the tumor microenvironment and have a substantial impact on the ineffectiveness of immunotherapy. Understanding the impact of these stressed factors on the tumor microenvironment could improve the efficacy of immunotherapy (92–95).

### Glioma stem cells

**Figure 3:**
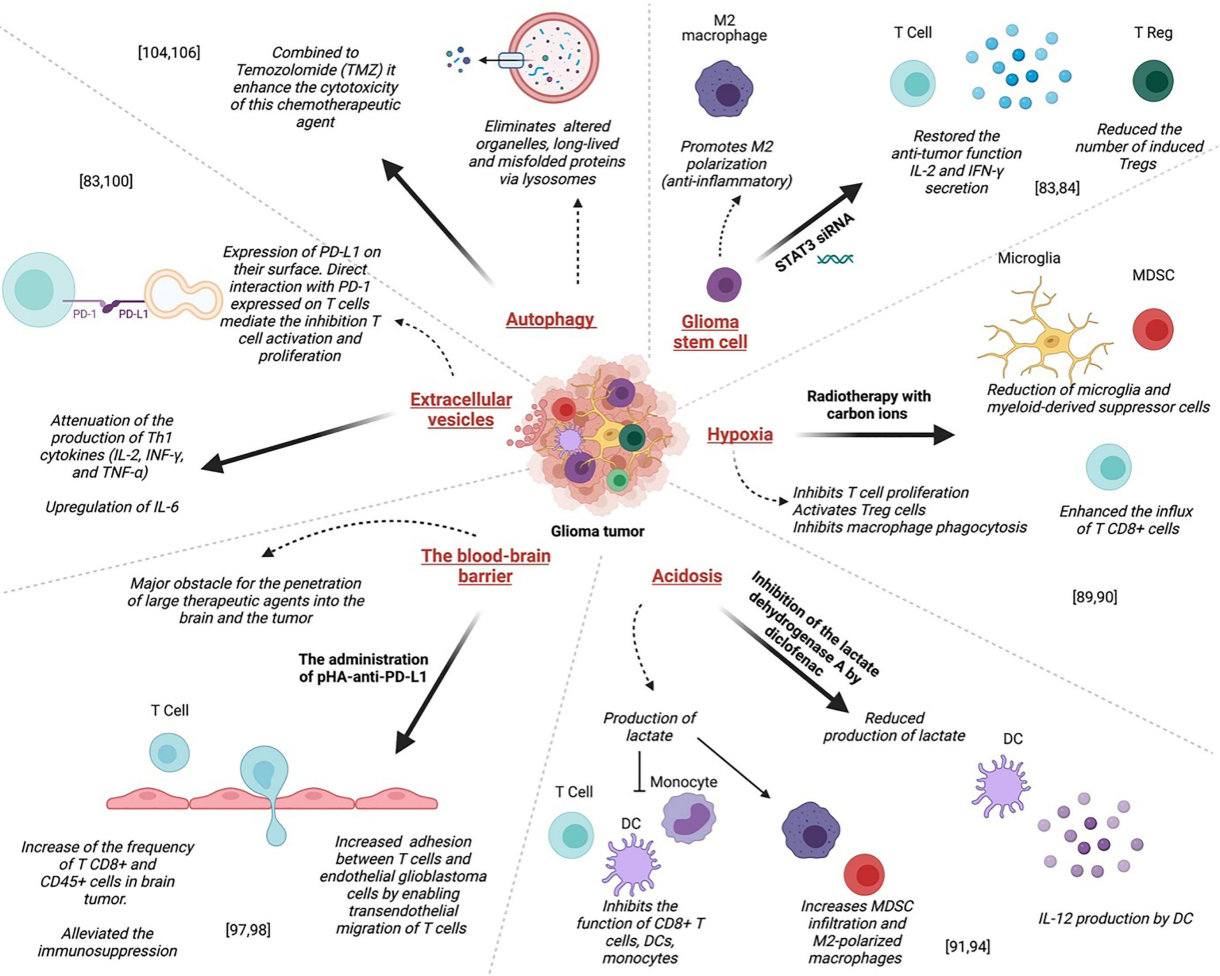
Mechanisms of resistance to glioma treatments: Left to right; Administration of pHA-anti-PD-L1 alleviated the immunosuppression, resulting in the infiltration of activated T cells in gliomas prolonged the survival of glioma-bearing mice. Direct interaction between PD-L1 expressed on glioma EV and PD-1 expressed on T cells mediate the inhibition of both CD4+ and CD8+ T cell activation and proliferation. Autophagy combined with Temozolomide (TMZ) could enhance the cytotoxicity of this chemotherapeutic agent. The blockade of the signal transducer and activator of transcription 3 (STAT3) in GSC by the STAT3 siRNA restored the anti-tumor function of T lymphocytes, inhibited their apoptosis, and reduced the number of induced Tregs. In GBM, hypoxia inhibits T cell proliferation and activation and, on the other hand, activates Treg cells. Acidosis inhibits the function of CD8+ T cells, DCs, and monocytes, and increases MDSC infiltration and M2-polarized macrophages that exhibit intense immunosuppressive function.

In the brain, stem cells exhibit large MHC antigens compared to normal neural stem cells; thus, immunotherapy using a vaccination technique to generate a T cell response, particularly against cancer stem cells, can be an effective therapeutic strategy (96). One of the main reasons for the resistance of glioma to therapies is the presence of a subpopulation of cells with self-renewal and tumor-initiating skills in the tumor microenvironment, namely glioma stem cells (GSCs) (93, 97). These cells are localized in niches where these are maintained as slowly dividing cells (93). The resistance is mainly due to its effect on the immune system as it promotes the polarization of macrophages towards the M2 phenotype and induces immune-suppressive activities through PD-L1 (98) (**Figure 3**). Interestingly, the blockade of the signal transducer and activator of transcription 3 (STAT3) in GSC, which is likely to be a key mediator of immune suppression, by the STAT3 siRNA restored the anti-tumor function (including IL-2 and IFN-γ secretion)of T lymphocytes, inhibited their apoptosis, and reduced the number of induced Tregs (99) (**Figure 3**).

So new approaches against glioma were built using the GSCs as targets; for example, 3-Bromopyruvate (3-BrPA) that is a glycolysis inhibitor and tumor energy blocker, was reported to inhibit the malignant phenotype of macrophages induced by glioma stem cells (100). There is also the transcription factor CCAAT/enhancer-binding protein delta (CEBPD), which regulates various genes responsive to inflammatory cytokines. This indicated that this was a key mechanism for GSC self-renewal in the inflammatory environment and exhibited a pivotal role in inducing the stem-like feature (101). Hence, targeting CEBPD seems an interesting therapeutic option to improve gliomas (101). In another study, it was shown that the stem cell marker CD44 is associated with poor prognosis and radiation resistance and the survival was improved in a glioma mouse model of using CD44−/− and CD44+/− mice compared to littermate controls (102).

### Hypoxia

Hypoxia corresponds to a low/insufficient O2 supply and constitutes a selection pressure that favors the rise of highly aggressive cells. Not only can these cells adapt to low O2 conditions, but these could also resist anti-cancer treatments (103).

In GBM, hypoxia inhibits T cell proliferation and activation and, on the other hand, activates Treg cells. Furthermore, it inhibits macrophage phagocytosis compared to normoxia conditions. This hypoxia-induced immunosuppressive effect was mediated via a signal transducer, activator of transcription 3(STAT3), hypoxia-inducible factor (HIF)-1α, and vascular endothelial growth factor (VEGF). Indeed, inhibition of these pathways, down-regulates this hypoxia-induced immunosuppressive effect (92) (**Figure 3**).

It was reported that radiotherapy with carbon ions could overcome several central glioma resistance mechanisms by elimination of hypoxic and stem cell-like tumor cells, but also through modulation of the glioma niche towards an anti-angiogenic and less immunosuppressive state. Radiation with carbon ions also reduced the recruitment of microglia and myeloid-derived suppressor cells, abrogated M2-like immune polarization, and enhanced the influx of CD8+ cells (104) (**Figure 3**).

### Acidosis

A common feature of glioma is acidosis caused by hypoxia. It is characterized by the accumulation of lactate and the decrease of pH (94)(**Figure 3**). It plays a critical role in glioblastoma chemoresistance by interfering with angiogenesis, apoptosis, oxidative stress, immune escape, and the activity of multidrug resistance (105). Aggressive brain tumors have been shown to produce lactate in their microenvironment, which helps them metastasize and evade the immune response and even radiation (106). It inhibits the function of CD8+ T cells, DCs, and monocytes, and increases MDSC infiltration and M2-polarized macrophages that exhibit intense immunosuppressive function (94). Chirasani et al. showed that inhibiting the lactate dehydrogenase A (LDHA) by diclofenac, is associated with a reduced production of lactate by glioma cells, which leads to IL-12 production by tumor-infiltrating DCs upon TLR stimulation (107) (**Figure 3**).

### The blood-brain barrier

The blood-brain barrier (BBB) in the context of brain tumors can be a major obstacle to the penetration of large therapeutic agents into the brain and the tumor. For example, the checkpoint inhibitor antibodies to cytotoxic T-lymphocyte-associated antigen 4 (CTLA-4) and programmed cell death-1 (PD-1) were unsuccessful, especially due to their inability to cross the BBB (108).

An interesting therapeutic way would be using nanoparticles to help antibodies cross the BBB. Indeed, using tumor-targeting immunoliposome nanocomplex encapsulating the tumor suppressor gene TP53 that is also known as SGT-53 (The p53 protein is involved in the regulation of cell cycle arrest and apoptosis in response to genotoxic and oncogenic stresses) in combination with anti-PD-1mAb resulted in the inhibition of tumor growth and induction of tumor cell apoptosis (109). This treatment also increased intratumoral T cell infiltration and suggested that SGT-53 can boost anti-tumor immunity and sensitize glioblastoma to anti-PD1 Ab treatment (109). Furthermore, a versatile drug carrier-poly (β-L-malic acid) (PMLA) was used to deliver covalently conjugated CTLA-4 and PD-1 antibodies by using the transferrin receptor (TfR)-mediated transcytosis to cross the BBB. This activated the local immune system in the brain tumor area through increased infiltration of CD8+ T cells, NK cells and macrophages and decreased infiltration of Treg cells (108).

A successful immunotherapy strategy for glioblastoma will ultimately consist of combinatorial therapy. It would allow enough tumor-specific T cells to enter and persist in an immune-permissive tumor microenvironment. Investigations could focus on the increase of adhesion between T cells and endothelial glioblastoma cells by enabling trans endothelial migration of T cells (110) (**Figure 3**). Such an approach would transform this deadly disease into an immunologically hot target. Guo et al. used an anti-PD-L1 conjugated with p-hydroxybenzoic acid (pHA) to facilitate the crossing of the BBB by the antibody through dopamine receptor-mediated transcytosis (110). This approach resulted in a significant increase in the frequency of CD8+ and CD45+ cells in brain tumors. The administration of pHA-anti-PD-L1 alleviated the immunosuppression, resulted in the infiltration of activated T cells in gliomas and prolonged the survival of glioma-bearing mice (111)(**Figure 3**). This indicates that immune checkpoint inhibitors, when adequately administered, could result in a positive outcome in GBM.

### Extracellular vesicles

Extracellular vesicles (EV) are small membrane vesicles derived from multivesicular bodies or bud from the plasma membrane. EVs released by glioma cells express PD-L1 on their surface (92). Direct interaction between PD-L1 expressed on glioma EV and PD-1 expressed on T cells mediate the inhibition of both CD4+ and CD8+ T cell activation and proliferation (95).

EV can also convert myeloid-derived innate immune cells to immune-suppressive or tumor-supporting effector cells. This results in the inhibition of T cell activation and tumor growth support through the secretion of specific cytokines (95)(**Figure 3**). Studies also demonstrated that EGFR+ EVs are useful diagnostic and prognostic markers of glioma. The expression of EGFR in serum EVs can accurately differentiate high-grade and low-grade glioma patients (112). Gabrusiewicz et al. reported that GBM-derived stem cell exosomes could induce upregulation of PD-L1 on CD14+ monocyte in human subjects. This results in the polarization of monocytes into immunosuppressive M2 macrophages (98). Another study found that GSC-derived exosomes significantly inhibited CD3+ T cell proliferation (113), decreased the expression of both activation markers, CD25 and CD69 (113) and attenuated the production of Th1 cytokines (IL-2, INF-γ, and TNF-α) while upregulated IL-6, aTh2 type cytokine (113). In GBM mouse model, EVs contribute to tumor growth and inhibited CD8^+^ T cell cytolytic activity (114).

### Autophagy

Autophagy is a catabolic mechanism that eliminates, via lysosomes, altered organelles, long-lived and misfolded proteins. It occurs in response to nutrient starvation or oxidative stress and leads to the formation of metabolic precursors (amino acids and fatty acids) and ATP, ensuring homeostasis and cell survival (115)(**Figure 3**). However, small repairs or major stress can lead to cell death, named autophagic death. It was shown that autophagy could modulate both invasion and resistance to therapy in GBM and that its inhibition (116), combined with Temozolomide (TMZ), could enhance the cytotoxicity of this chemotherapeutic agent (117)(**Figure 3**). It would be interesting to explore further the relationship between autophagy and the modulation of the immune system in glioma for a better understanding of resistance mechanisms to therapies.

## Concluding remarks

In the present review, we provided evidence indicating that the immune system is indeed involved in glioma physiopathology. However, the immune components are strongly inhibited in the tumor microenvironment through distinct mechanisms including immune checkpoints, hypoxia, acidosis, glioma stem cells, and extracellular vesicles. The BBB constitutes a major obstacle behind the failure of immunotherapy in gliomas. Therefore, we suggest further studies involving all these different facets of the glioma microenvironment. Lastly, in order to potentially benefit from current and future immunotherapies, investigations in glioma should decipher adequate ways to facilitate BBB crossing of these therapeutic agents.

## Author contributions

SR conceived and designed, did the literature search, writing of the manuscript, and made the figures; SK writing of the manuscript and made the figures; AG made the figures; ON, KR, and RU revised the manuscript. AB: supervised, corrected, reviewed, and edited the manuscript. All authors contributed to the article and approved the submitted version.
